# Log odds of negative lymph nodes/T stage ratio (LONT): A new prognostic tool for differentiated thyroid cancer without metastases in patients aged 55 and older

**DOI:** 10.3389/fendo.2023.1132687

**Published:** 2023-03-22

**Authors:** Xuezhen Wang, Yufan Wu, Xiaoxia Li, Jinsheng Hong, Mingwei Zhang

**Affiliations:** ^1^ Department of Radiotherapy, Cancer Center, The First Affiliated Hospital of Fujian Medical University, Fuzhou, China; ^2^ Department of Radiotherapy, National Regional Medical Center, Binhai Campus of the First Affiliated Hospital, Fujian Medical University, Fuzhou, China; ^3^ Key Laboratory of Radiation Biology of Fujian Higher Education Institutions, The First Affiliated Hospital, Fujian Medical University, Fuzhou, China

**Keywords:** DTC, LONT, negative lymph nodes, negative lymph nodes/T stage, prognosis

## Abstract

**Background:**

The optimal approach to assess the postoperative status of lymph nodes in differentiated thyroid cancer (DTC) remains controversial. Our aim was to determine if the log odds of negative lymph nodes/T stage ratio (LONT) could serve as a new prognostic and predictive tool for DTC without metastases in patients aged ≥ 55 years.

**Methods:**

The Surveillance, Epidemiology, and End Results (SEER) database was used to study the role of LONT in patients aged ≥55 years diagnosed with DTC without metastases. The primary outcome was overall survival (OS). The Kaplan-Meier method and the Cox proportional hazard regression model were used to calculate the outcome. Moreover, the robustness of research findings was evaluated using sensitivity analyses.

**Results:**

A total of 21,172 DTC patients aged ≥55 years without distant metastasis were enrolled. Multivariate Cox regression analyses and a “floating absolute risk” analysis showed that a LONT ≥0.920 (vs. -0.56 to 0.92) was a protective factor for OS in DTC patients. Sensitivity analyses revealed an E-value of 1.98 for the obtained LONT value. In subgroup analyses, LONT was correlated significantly with OS in different subgroups of negative lymph nodes, stage-I–II subgroups and the N0 subgroup. The conditional probability of survival of DTC improved with prolonged survival time in the LONT ≥0.920 group.

**Conclusion:**

A high LONT was associated with longer OS compared with low LONT in patients aged ≥55 years with non-metastatic DTC. LONT could provide valuable information for undertaking postoperative evaluations.

## 1 Introduction

Thyroid cancer (TC) is the most common malignant tumor of the endocrine system. According to the origin of the tumor and the variability in differentiation, the pathologic type of >90% of TCs is differentiated thyroid cancer (DTC), including papillary thyroid carcinoma (PTC) and follicular thyroid carcinoma (FTC) (1). Thyroidectomy and standardized dissection of lymph nodes in the neck are the first-line treatments for DTC (2, 3).

Tumor-node-metastasis (TNM) staging for TC is important because it is used to guide the treatment and prognosis of patients. However, this system focuses only on the depth of tumor invasion, number of lymph-node metastases, and presence of distant metastases, while prognoses vary even among patients with TC at identical stages. Therefore, developing a more intricate and detailed method of prognostic evaluation may help to accurately predict the outcome, and choose a more targeted and rational individualized treatment protocol.

The main factors influencing DTC outcomes are age and TNM stage. Age is an independent risk factor for disease-specific survival (DSS) in TC patients (4, 5). The eighth version of the Thyroid Cancer Staging System set by the American Joint Committee on Cancer (AJCC) continues to adopt anatomic staging based on T, N and M. The diagnostic cutoff point for age required for the prognosis increases from 45 to 55 years (6). Metastasis in the lymph nodes in the neck is a risk factor for recurrence and shortened survival in patients with TC. However, the strategy for dissection of these lymph nodes is controversial (7–11). Often, dissection of regional lymph nodes for DTC is based on individualized treatment with varying degrees of dissection, which challenges the objective assessment of the postoperative status of lymph nodes. With respect to lymph-node management in DTC, postoperative evaluation of lymph-node status is influenced by multiple factors such as the surgical approach. The number of positive lymph nodes, examination of lymph nodes (ELN) and the number of negative lymph nodes (NLN) are classical evaluation indicators of lymph-node status, which are associated with survival in gastric (12), colorectal (13, 14), and breast cancer (15).

Log odds of negative lymph nodes/T stage ratio (LONT) refers to information on the disease stage and is used to quantify the degree of lymph-node dissection. NLNs represent the total level of lateral neck dissection (LND) and the T stage represents disease severity. NLNs adjusted according to the T stage denote the relative number of NLNs removed from each patient. A high value of LONT indicates more NLNs obtained, while a low LONT value means that fewer NLNs were obtained. Therefore, LONT can be used to compare the relative levels of LND among patients. Different TNM stages, ELNs, or NLNs, and the same LONT denote the same risk level (16). Studies have shown that risk stratification using LONT in patients after surgery for gastric cancer reflects disease severity and integrates the prognostic information of the degree of lymph-node dissection, while being closely related to the treatment outcome (16). However, studies using LONT to investigate TC are lacking, and the prognostic value of LONT in TC patients remains unknown.

To address this, we retrospectively analyzed clinical-pathology data in 21,172 patients with DTC who underwent resection based on the Surveillance, Epidemiology, and End Results (SEER) database of the US National Cancer Institute (Bethesda, MD, USA). We aimed to investigate the effect of LONT on the prognosis of DTC patients aged ≥55 years without metastasis at the first visit using the Kaplan–Meier method, and a Cox regression analysis. Moreover, the robustness of research findings was evaluated by sensitivity analysis and calculation of E-values. This is the minimum strength of association (risk ratio scale) that an unmeasured confounder would need to have with both exposure and the outcome to fully explain the specific exposure-outcome association.

## 2 Materials and methods

### 2.1 Data source and cohorts

A population-based study was undertaken retrospectively using data from the SEER database using SEER*Stat 8.3.9. From 2010 to 2015, patients aged 55 and older diagnosed with non-metastatic DTC were evaluated. The exploration was based on the SEER database, which discloses the demographic features, clinicopathological characteristics, and survival information of patients. As the data were derived from anonymous public databases, ethical approval was not sought or needed.

The inclusion criteria were: (1) diagnosis of TC without metastasis; (2) age ≥55 years; (3) histology type included International Classification of Diseases for Oncology, third edition (ICD-O-3) code 8050/3 (papillary carcinoma, not otherwise specified (NOS)), 8260/3 (papillary adenocarcinoma, NOS), 8290/3 (oxyphilic adenocarcinoma), 8330/3 (follicular adenocarcinoma, NOS), 8331/3 (follicular adenocarcinoma, well differentiated), 8335/3 (follicular carcinoma, minimally invasive), 8340/3 (papillary carcinoma, follicular variant), 8341/3 (papillary microcarcinoma), 8342/3 (papillary carcinoma, oxyphilic cell), 8343/3 (papillary carcinoma, encapsulated), and 8344/3 (papillary carcinoma, columnar cell); (4) history of total thyroidectomy.

The exclusion criteria were: (1) unknown number of examined or positive regional lymph nodes; (2) unknown T, N, and M stages; (3) incomplete information on age, sex, ethnicity, tumor diameter, survival, and vital signs; (4) T0 and T4 stages.

### 2.2 Definition of LONT

“LONT” is defined as the log odds of (negative lymph nodes +1)/T stage ratio. The description of LONT calculation is as follows:


$$log^{\left(NLNs+1\right)/T\;stage}$$

T stages T1a, T1b, T2, T3, T4a, and T4b are assigned as 1, 2, 3, 4, 5, and 6, respectively. NLNs denote the ELNs minus the count of positive lymph nodes. The value of one is added for NLNs to avoid the occurrence of zero. A restricted cubic spline was used to assess the association between LONT and survival to avoid assuming it was a simple linear association. LONT was categorized if non-linearity was detected. The optimal thresholds for LONT were determined on the result of restricted cubic spline.

### 2.3 Selection of variables

The main variables extracted from the SEER database were “age at the diagnosis”, “sex”, “ethnicity”, “marital status at the diagnosis”, “year of the diagnosis”, “stage group, derived from AJCC seventh edition (2010–2015)”, “T stage derived from AJCC seventh edition (2010–2015)”, “N stage derived from AJCC seventh edition (2010–2015)”, “M stage derived from AJCC seventh edition (2010–2015)”, “tumor grade”, “radiotherapy recode”, “chemotherapy recode”, “CS tumor size”, “first indicator of primary malignancy”, “SEER cause-specific death classification”, “survival in months”, “vital signs”, and “histology type (based on ICD-O-3)”. The histology type includes papillary carcinoma (PC), follicular adenocarcinoma (FC), papillary carcinoma, follicular variant (PCFV), Papillary microcarcinoma (PMC).

### 2.4 Statistical analyses

Categorical demographic (ethnicity, sex) and clinical data was analyzed by the chi-square test. The designated outcome was overall survival (OS). Survival curves were constructed using the Kaplan-Meier method. The log-rank test was undertaken to compare various subgroups. Univariate and multivariate Cox proportional hazards tests were conducted for OS using the “survival” package in R 4.0.1 (R Institute for Statistical Computing, Vienna, Austria). For comparisons involving multiple groups, 95% confidence intervals (CIs) were calculated using the floating absolute risks (FAR) method to enable valid comparisons between any two groups and not only with the reference group (17). Stratified analyses were conducted with Cox models; hazard ratios (HR), and 95% CIs were calculated for subgroups. The “conditional probability of survival” was defined as the probability of surviving to “Y” years after the diagnosis given survival to “X” (X< Y) years. Additionally, it can describe in detail the survival of patients at different time stages (18). Further analyses of E-value sensitivity were conducted to evaluate the robustness of the association to unmeasured confounding using the “E-Value” package within R. Statistical analyses were also conducted employing R. P < 0.05 (two-tailed) was considered significant.

## 3 Results

### 3.1 The relationship between LONT and survival

The restricted cubic spline curve showed that LONT had a nonlinear association with survival. HR for death was lowest near the LONT of -0.586, with a gradual rise after 0.920 (**Figure S1**). Then, optimal LONT thresholds were identified and the cohort was separated into three groups: LONT< -0.586, -0.586≤ LONT<0.920, and LONT ≥0.920.

### 3.2 Baseline population demographics

A total of 21,172 patients aged ≥55 years diagnosed with TC without metastasis between 2010 and 2015 were enrolled. There was no significant difference between the three groups in the distribution of sequence number and multiple primary tumors. Detailed information about the demographic and clinicopathological characteristics of patients is shown in **Table 1**.

**Table 1:** Demographic and clinicopathologic characteristics of the study cohort.

Variables	Total (n = 21172)	LONT< -0.586 (n = 7343)	0.586≤LONT< 0.920 (n = 9911)	LONT≥0.920 (n = 3918)	p
Age, n (%)					< 0.001
55~65	12592(59)	4101(56)	5954 (60)	2537 (65)	
66~74	5919 (28)	2065(28)	2868 (29)	986 (25)	
≥75	2661 (13)	1177(16)	1089 (11)	395 (10)	
Sex, n (%)					< 0.001
Female	15097(71)	4912(67)	7417 (75)	2768 (71)	
Male	6075 (29)	2431(33)	2494 (25)	1150 (29)	
Race, n (%)					< 0.001
Black	1433 (7)	598 (8)	694 (7)	141 (4)	
Others	2281 (11)	887 (12)	978 (10)	416 (11)	
White	17458(82)	5858(80)	8239 (83)	3361 (86)	
Marital status, n (%)					0.001
Divorced/separated	2073 (10)	710 (10)	999 (10)	364 (9)	
Married	13334(63)	4512(61)	6266 (63)	2556 (65)	
Single/Unmarried	2386 (11)	876 (12)	1085 (11)	425 (11)	
Widowed/Others	3379 (16)	1245(17)	1561 (16)	573 (15)	
Diagnosis, n (%)					0.026
2010	2995 (14)	1101(15)	1391 (14)	503 (13)	
2011	3341 (16)	1182(16)	1578 (16)	581 (15)	
2012	3431 (16)	1161(16)	1622 (16)	648 (17)	
2013	3717 (18)	1235(17)	1770 (18)	712 (18)	
2014	3812 (18)	1306(18)	1788 (18)	718 (18)	
2015	3876 (18)	1358(18)	1762 (18)	756 (19)	
AJCC, n (%)					< 0.001
I	11006(53)	2223(31)	6885 (70)	1898 (49)	
II	2606 (12)	1568(22)	864 (9)	174 (5)	
III	5318 (25)	2995(41)	1610 (16)	713 (19)	
IV	2020 (10)	491 (7)	465 (5)	1064 (28)	
AJCC.T, n (%)					< 0.001
T1a	7805 (37)	0 (0)	5864 (59)	1941 (50)	
T1b	4642 (22)	2392(33)	1499 (15)	751 (19)	
T2	3072 (15)	1666(23)	1063 (11)	343 (9)	
T3	4886 (23)	2918(40)	1270 (13)	698 (18)	
T4a	552 (3)	261 (4)	153 (2)	138 (4)	
T4b	215 (1)	106 (1)	62 (1)	47 (1)	
AJCC.N, n (%)					< 0.001
N0	17156(81)	6421(87)	8530 (86)	2205 (56)	
N1	4016 (19)	922 (13)	1381 (14)	1713 (44)	
Grade, n (%)					< 0.001
I~II	5183 (24)	1890(26)	2320 (23)	973 (25)	
III~IV	274 (1)	137 (2)	78 (1)	59 (2)	
Unknown	15715(74)	5316(72)	7513 (76)	2886 (74)	
Tumor Size, n (%)					< 0.001
~1cm	7404 (35)	283 (4)	5421 (55)	1700 (43)	
1~2cm	6424 (30)	2914(40)	2303 (23)	1207 (31)	
2~4cm	4917 (23)	2542(35)	1663 (17)	712 (18)	
4+cm	2427 (11)	1604(22)	524 (5)	299 (8)	
Hist Type, n (%)					< 0.001
PC	11216(53)	3387(46)	5348 (54)	2481 (63)	
FC	950 (4)	669 (9)	235 (2)	46 (1)	
PCFV	7170 (34)	2660(36)	3407 (34)	1103 (28)	
PMC	788 (4)	48 (1)	597 (6)	143 (4)	
Others	1048 (5)	579 (8)	324 (3)	145 (4)	
Sequence Number, n (%)					0.309
NO	17006(80)	5856(80)	7993 (81)	3157 (81)	
YES	4166 (20)	1487(20)	1918 (19)	761 (19)	
Multiple Primary Tumors, n (%)					0.234
NO	15444(73)	5304(72)	7269 (73)	2871 (73)	
YES	5728 (27)	2039(28)	2642 (27)	1047 (27)	
NLN, n (%)					< 0.001
0	11254(53)	6538(89)	4716 (48)	0 (0)	
1~8	7829 (37)	805 (11)	5098 (51)	1926 (49)	
≥9	2089 (10)	0 (0)	97 (1)	1992 (51)	
Chemotherapy, n (%)					< 0.001
NO	21100(100)	7312(100)	9893 (100)	3895 (99)	
YES	72 (0)	31 (0)	18 (0)	23 (1)	
Radiation, n (%)					< 0.001
NO&Unknown	11061(52)	2743 (37)	6266 (63)	2052 (52)	
YES	10111(48)	4600 (63)	3645 (37)	1866 (48)	

### 3.3 OS

**Figure 1:**
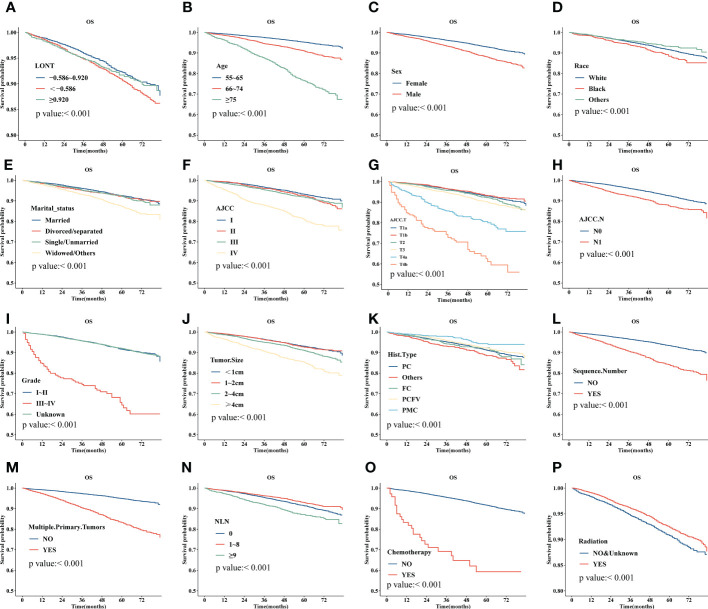
Kaplan–Meier plot for overall survival (OS) for LONT **(A)**, age **(B)**, sex **(C)**, ethnicity **(D)**, marital status **(E)**, stage (based on AJCC system) **(F)**, T stage (based on AJCC system) **(G)**, N (based on AJCC system) **(H)**, tumor grade **(I)**, tumor diameter **(J)**, histology type **(K)**, sequence number **(L)**, multiple primary tumors **(M)**, NLNs **(N)**, chemotherapy **(O)**, and radiotherapy **(P)** of patients with differentiated thyroid cancer without metastases aged 55 years and older.

**Figure 2:**
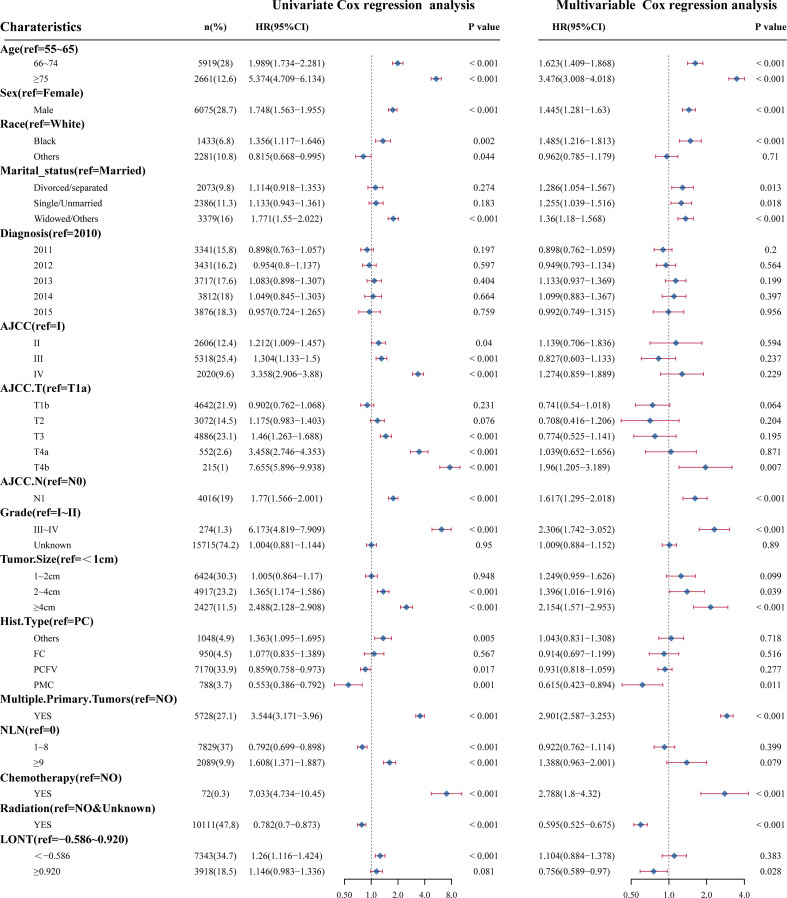
Univariate Cox regression analyses and multivariate Cox regression analyses for LONT and clinical features. Forest plot of hazard ratios demonstrating the results of Cox regression analyses. The dashed line denotes where HR = 1.

Survival analyses using the Kaplan-Meier method revealed a significantly poor OS (P < 0.001) for patients with low LONT (**Figure 1**). Univariate and multivariate analyses were undertaken using data from all patients to assess the potential prognostic factors. In the univariate Cox regression analysis, the following factors were associated with shortened OS: age groups of 66–74 years and >75 years (*vs*. 55–65 years), male sex, black ethnicity (*vs*. white ethnicity), marital status of widowed/other (*vs*. married), grade of II, III, or IV (*vs*. I), stage of T2, T3, T4a, or T4b (*vs*. Ia), N1 stage (*vs*. N0), grade of III–IV (*vs*. I–II), tumor diameter of 2–4 cm or >4 cm (*vs*.<1 cm), multiple primary tumors, and high NLNs. LONT >0.920 was an independent protective factor for OS (HR, 0.756; 95%CI, 0.589–0.97, P =0.028) based on multivariate Cox regression analyses after adjustment for baseline demographic and clinical features (**Figure 2**). The floating absolute risk method provides the variance in the logarithm of the hazard ratio for each category (including the reference category) to facilitate comparisons among the different LONT categories (**Table 2**). Compared with the OS values of the -0.586 to 0.920 group, the adjusted HR for LONT >0.920 was 0.756 (95%CI, 0.589–0.97). Stratified analyses showed the HRs for OS across two LONT groups stratified by demographic and clinicopathologic features (**Figure S2**). The association between LONT and OS remained significant for male sex (P =0.026), black ethnicity (P =0.033), marital status divorced/separated (P =0.015), T4b stage (P =0.005), N status (P<0.001), grade of III–IV (P<0.001), and tumor diameter >4 cm (P<0.001).

**Table 2:** Adjusted Hazard Ratios for OS to patients with different LONT by floating absolute risk methods.

	LONT		
	0.586≤ LONT< 0.920 (n = 9911)	LONT≥ 0.920 (n = 3918)	LONT< -0.586 (n = 7343)
Hazard ratio	1	0.756	1.104
95% CI, with floating absolute risk	–	0.589-0.970	0.884-1.378
95% CI, without floating absolute risk	–	0.577-0.991	0.864-1.411

### 3.4 Sensitivity analyses

Analyses undertaken using the FAR method yielded results similar to multivariate Cox regression analyses for OS. Sensitivity analyses for E-values were also conducted to evaluate the robustness of the association to unmeasured confounding (**Figure S3**). E-values (95% CI) were calculated for LONT ≥0.920 (*vs*. -0.586 to 0.920), 75 years of age and 66–74 years (*vs*. 55–65 years), sex, black ethnicity (*vs*. white ethnicity), married marital status (*vs*. other), multiple primary tumors, tumor diameter of >4 cm and 2–4 cm (*vs*.<1 cm), histology type of papillary microcarcinoma (*vs*. papillary carcinoma, NOS, and papillary adenocarcinoma, NOS), grade of III–IV (*vs*. I–II), T4b stage (*vs*. T1a stage), N status, chemotherapy, and radiotherapy. The respective values were as follows:1.976(1.209-2.786), 6.410(5.466-7.5), 2.629(2.168-3.141), 2.247(1.881-2.643), 2.334(1.728-3.027), 2.06(1.641-2.512), 1.892(1.293-2.51), 1.821(1.24-2.4), 5.249(4.613-5.96), 3.731(2.518-5.355), 2.140(1.143-3.241), 2.635(1.483-4.16), 4.041(2.879-5.555), 5.021(3-8.107), 3.332(1.702-5.831), 2.616(1.913-3.451), 5.021(3-8.107), and 2.750(2.326-3.218).

### 3.5 Conditional probability of survival

**Figure 3:**
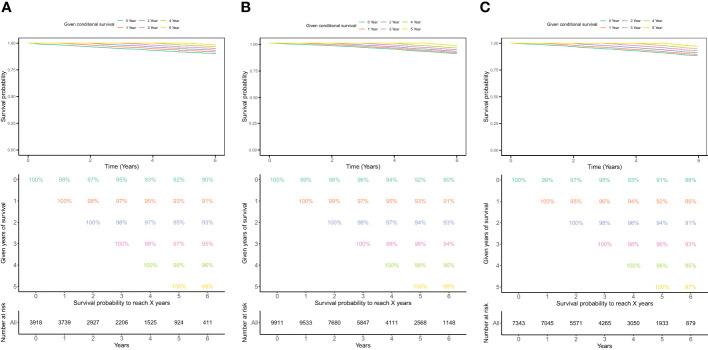
The conditional probability of survival of LONT ≥0.920 **(A)**, -0.586≤ LONT<0.920 **(B)**, and LONT<-0.586 **(C)**.

**Figure 3** shows the conditional probability of survival at various time points for the study cohort combining LONT< -0.586 (**Figure 3A**), -0.586≤ LONT<0.920 (**Figure 3B**), and LONT ≥0.920 (**Figure 3C**) separately. In the LONT ≥0.920 group, the annual conditional probability of survival increased with OS. From a 92% chance of survival immediately after the diagnosis, the probability of OS at 1, 2, 3, and 4 years after the diagnosis increased by 93%, 95%, 97%, and 98%, respectively. The probability of OS the following year decreased from 98% to 96% at 3 years and 5 years, respectively.

## 4 Discussion

The TNM staging system contains only certain characteristic indicators of tumors. For example, lymph-node status is one of the most important predictors for patients with DTC. We comprehensively analyzed the information on the number of NLNs and tumor stage after surgery. LONT was proposed as an indicator to quantify the degree of lymph-node dissection and disease severity intraoperatively and to predict the survival of patients with DTC. We undertook retrospective data analyses of 21,172 patients with DTC aged ≥55 years without distant metastasis. Multivariate COX regression analyses and FAR analyses showed that LONT ≥0.920 (*vs.* -0.56 to 0.92) was a protective factor for OS in patients with DTC. Sensitivity analyses demonstrated that with an E-value of 1.98 for LONT ≥0.920 (vs. -0.56 to 0.92), the results were stable even in the presence of unmeasured confounding factors. Subgroup analyses revealed that LONT correlated significantly with OS in different NLN subgroups, stage-I–II subgroups, and the N0 subgroup. The conditional probability of survival of patients with DTC improved with a prolonged survival time in the LONT ≥0.920 group.

Furthermore, an accurate prognosis for patients with TC s crucial for the postoperative treatment and follow-up plan. The TNM staging system set by the AJCC is the most widely used system for prognostic assessment. Age at TC diagnosis is an independent predictor of DSS (19, 20). The eighth version of the AJCC staging system for TC continues to adopt anatomic staging based on T, N and M. However, a cutoff for the age at the DTC diagnosis from 45 to 55 years is the best single time point for the prognostic model (4–6, 21). Patients with DTC aged ≥55 years with stage-I–IVa disease were included in our study; their prognosis is quite heterogeneous, with 10-year DSS fluctuating between 50% and 100% according to data from the AJCC staging system. These patients may achieve optimal surgical results and longer OS after additional treatment, but older patients with DTC tend to seek medical treatment late and often have comorbidities. Therefore, the risk of metastasis and disease recurrence is higher in older patients. Certain studies have found that survival in DTC patients aged<55 years is similar to that in patients with stage-III TC, and significantly lower than that in patients aged ≥55 years with stage-II TC (22, 23).

In the present study, age remained an independent predictor in the multivariate Cox regression mode. Older patients with DTC had to be stratified further according to the diameter of the primary tumor and lymph-node status. The location of metastatic lymph nodes does not affect the prognosis of older patients, while other indicators such as the number and maximum diameter of the primary tumor, maximum diameter of metastases, and external invasion of lymph nodes have been postulated as potential prognostic factors (24, 25).

Studies have shown (26–28) that the prognosis of patients with N1b-stage disease is significantly worse than that of patients with N1a-stage disease. Metastasis to lateral-neck lymph nodes is an independent risk factor for death. Moreover, indicators related to lymph-node metastasis are associated with outcomes in patients with DTC (29–31). The number of lymph nodes and the number of NLNs may reflect the degree of lymph-node dissection required. Certain studies have suggested that with greater ELNs (32) or NLNs (33), fewer micro-metastases will be missed in the lymph nodes. ELNs and NLNs are independent prognostic factors for lung (34), breast (35), and esophageal cancer (32). However, whether there are differences in DTC outcomes for different combinations of lymph-node metastasis-related indicators remains unknown.

The predictive value of tumor diameter-related parameters is controversial, but most scholars agree that a large primary tumor diameter is a clinicopathologic risk factor associated with poor DTC outcomes (36). The tumor diameter was<2 cm in 65% of patients in the present study. One meta-analysis (36) showed that patients with a tumor diameter between 1 cm and 2 cm had a greater risk of postoperative recurrence and death compared to that in patients with a tumor diameter<1 cm. Bilimoria et al. (37) undertook a retrospective study involving 52,173 patients with PTC. Compared with those who underwent total thyroidectomy, patients with a tumor diameter 1–2 cm had insufficient initial treatment, suffered recurrence, and had a poor prognosis. In the AJCC staging system, tumor diameter is not completely equivalent to the T stage, which integrates tumor diameter and gross extrathyroidal extension. Song and colleagues performed a study involving 3,104 patients undergoing thyroid surgery. The DSS of patients with gross extrathyroidal extension was significantly lower than that of patients with stage-T3 disease (38). The authors suggested adjusting the classification of patients with tumor diameter ≤4 cm from stage T3b toT2 to obtain more accurate survival predictions.

In our study, LONT comprised the number of lymph-nodes examined, the number of positive lymph nodes, and the T stage, which integrated the details of tumor diameter and gross extrathyroidal extension. LONT reflected the disease status after surgery and provided information on the degree of lymph-node dissection. In TC, the information required for complete staging may not be available for perioperative thyroidectomy. The eighth version of TNM staging by the AJCC states that information obtained within four months after the surgical procedure can be used to update T, N, and M stages. It also promotes clinical application of LONT and provides additional data for the individualized diagnosis and treatment of TC.

The current study is the first to apply LONT to quantify the relative degree of lymph-node dissection. LONT provides information on postoperative lymph-node dissection and T stage. The prognostic evaluation of patients with M0 disease and identical ELNs or NLNs is a powerful tool. The pattern of lymph-node metastasis varies among different pathologic types. More than 90% of DTC is PTC. Compared with FTC, metastasis to lymph nodes in the neck in PTC is more common and may often occur early. In one large-cohort study, the prevalence of lymph-node metastasis in PTC cases was ≤22% (39). Treatment of metastases in lymph nodes in the lateral neck in patients with DTC remains controversial. With respect to analyses of efficacy and economic perspectives, prospective studies to demonstrate the impact of different surgical scopes on the prognosis are difficult to conduct. Glover et al. (24) stated that safe implementation of prophylactic central neck dissection by an experienced surgeon can compensate for the unreliability of preoperative and intraoperative assessments of lymph-node metastasis in the central compartment. This approach can avoid the regional recurrence caused by insufficient dissection due to a narrow surgical scope and obtain more accurate PTC stages (40), reduce postoperative thyroglobulin levels, reduce the requirement for reoperation (41), reduce the risk of recurrence, and improve the OS (42). However, some studies have shown occult lymph-node metastases have a limited impact upon survival and recurrence (9). Prophylactic LND in patients with negative clinical lymph-node metastasis (cN0) expands the surgical scope and increases the risk of injuring blood vessels, nerves, and lymphatic vessels in the lateral neck (43). *Guidelines for the Diagnosis and Treatment of Adult Thyroid Nodules and Differentiated Thyroid Cancer* (44) published by the American Thyroid Association in 2015 recommend that conventional therapeutic LND of lymph nodes should not be carried out for patients with cN0 disease. Moreover, therapeutic LND should be undertaken for patients with DTC exhibiting lateral-neck-lymph node metastases (cN1b) by clinical and/or preoperative and intraoperative assessments. In contrast, this study proposes LONT as a new prognostic index, which does not consider the surgical approach/procedure, thus avoiding the impact of different surgical methods, while focusing on postoperative outcomes. Subgroup analyses in the present study showed a survival benefit with high LONT in patients with stage-I–II disease and N0 status. Hence, disease severity should not be underestimated in low-risk patients, and follow-up monitoring must be strengthened.

In addition, we revealed that high LONT prolonged OS significantly through adjustment of demographic and clinicopathologic factors. A high LONT value suggested a significant survival benefit. Specifically, higher the LONT values reflected, lower T stages, and fewer lymph nodes involved. Stroup and colleagues (45) retrospectively evaluated 20,513 women with DTC aged ≥40 years. They found OS and DSS to be shorter in the “regional” group (tumor confined to the thyroid gland and soft tissue) than in the “localized” group (confined to the thyroid gland). The difference in life expectancy of patients with DTC (pT1–3, pN0–1, M0) is not significant compared with that in the treated general population (46). The reason for this observation may be that the long-term survival of TC patients has been improved along with the screening and early treatment of TC. Furthermore, most studies have not distinguished subgroups by age.

Nevertheless, our study had several limitations. First, despite being based on a large cohort, our study was limited by its retrospective nature. Second, there was a selection bias for patients with TC. Third, LONT accounts for the degree of lymph-node dissection but cannot fully evaluate the success of surgical treatment or provide information on complications. Fourth, information on subsequent treatment, such as specific regimen and dose of radiotherapy and chemotherapy was not available. Fifth, the population consisted of middle-aged and older patients (≥55 years). Therefore, the results cannot be generalized to younger patient groups. Sixth, whether patients received targeted therapy or immunotherapy based on the molecular properties of TC was unknown, as we did not possess the information on their biomolecular markers. Finally, the postoperative pathology data on vascular invasion, nerve invasion, and condition of the incisal edge were not available.

## 5 Conclusions

LONT is a new prognostic indicator reflecting the relative degree of LND in different patients. It can predict the OS in patients aged ≥55 years without distant metastases undergoing surgical treatment regardless of preoperative and intraoperative outcomes. It could provide more valuable information for clinicians to conduct postoperative evaluations.

## Data availability statement

The raw data supporting the conclusions of this article will be made available by the authors, without undue reservation.

## Author contributions

XW: Writing – original draft (lead); writing – review and editing (equal input); formal analysis (equal input). YW: Visualization (equal input); formal analysis (equal input). XL: Visualization (equal input); formal analysis (equal input). JH: Writing – review and editing (equal input). MZ: Conceptualization (lead); writing – review and editing (equal input). All authors contributed to the article and approved the submitted version.

## Supplementary material

Supplementary Figure 1 Association of LONT with OS.

Supplementary Figure 2 Subgroup analyses for LONT.

Supplementary Figure 3 E-value of LONT and clinical features in sensitivity analyses.
