# Supplementary figures and images for: Log odds of negative lymph nodes/T stage ratio (LONT): A new prognostic tool for differentiated thyroid cancer without metastases in patients aged 55 and older
Source: Front Endocrinol (Lausanne). 2023 Mar 22;14:1132687. doi: 10.3389/fendo.2023.1132687 (PMC10073738; doi:10.3389/fendo.2023.1132687)

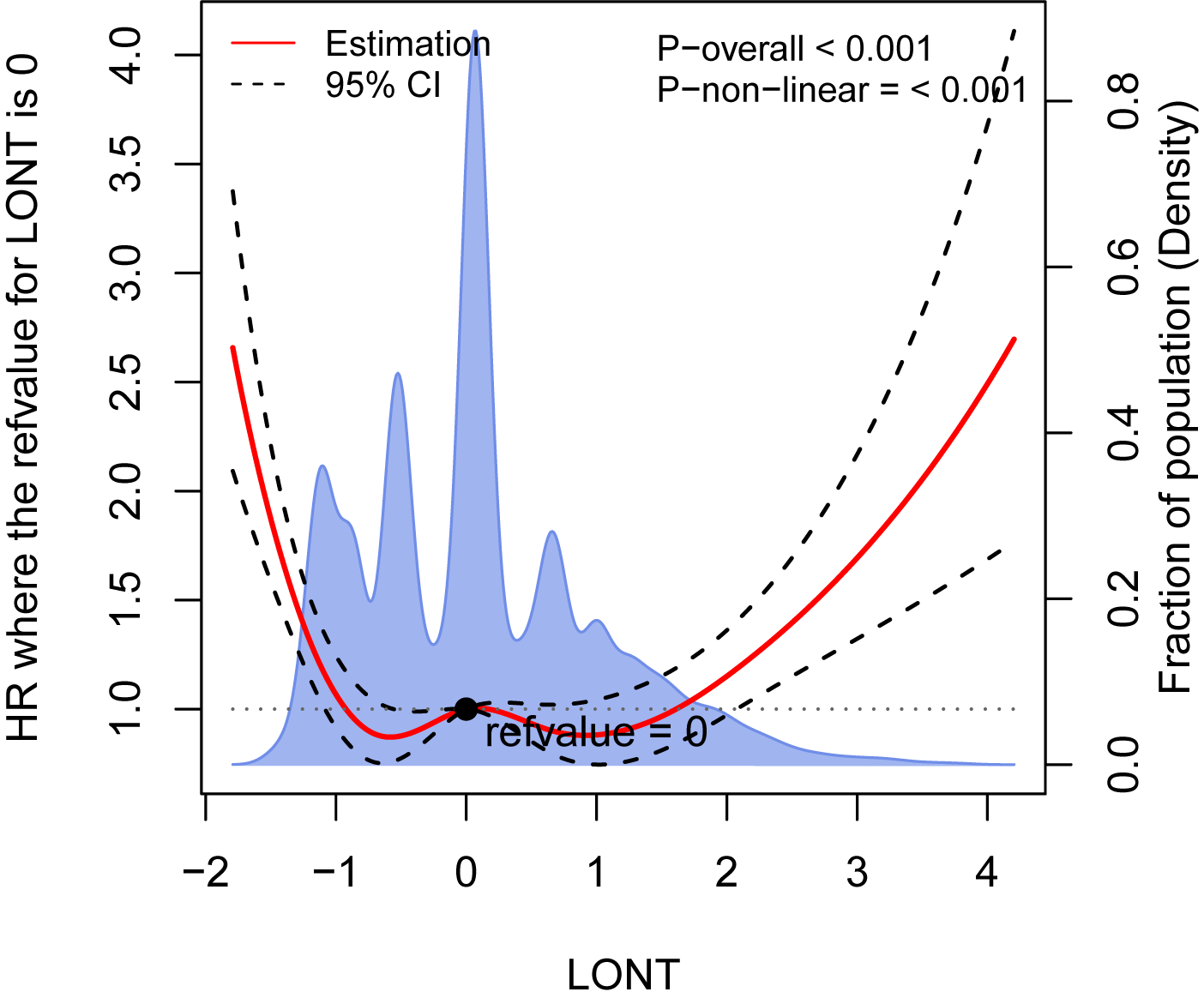

Supplement: Supplementary Figure 1 — Association of LONT with OS. [file Image_1.tif]

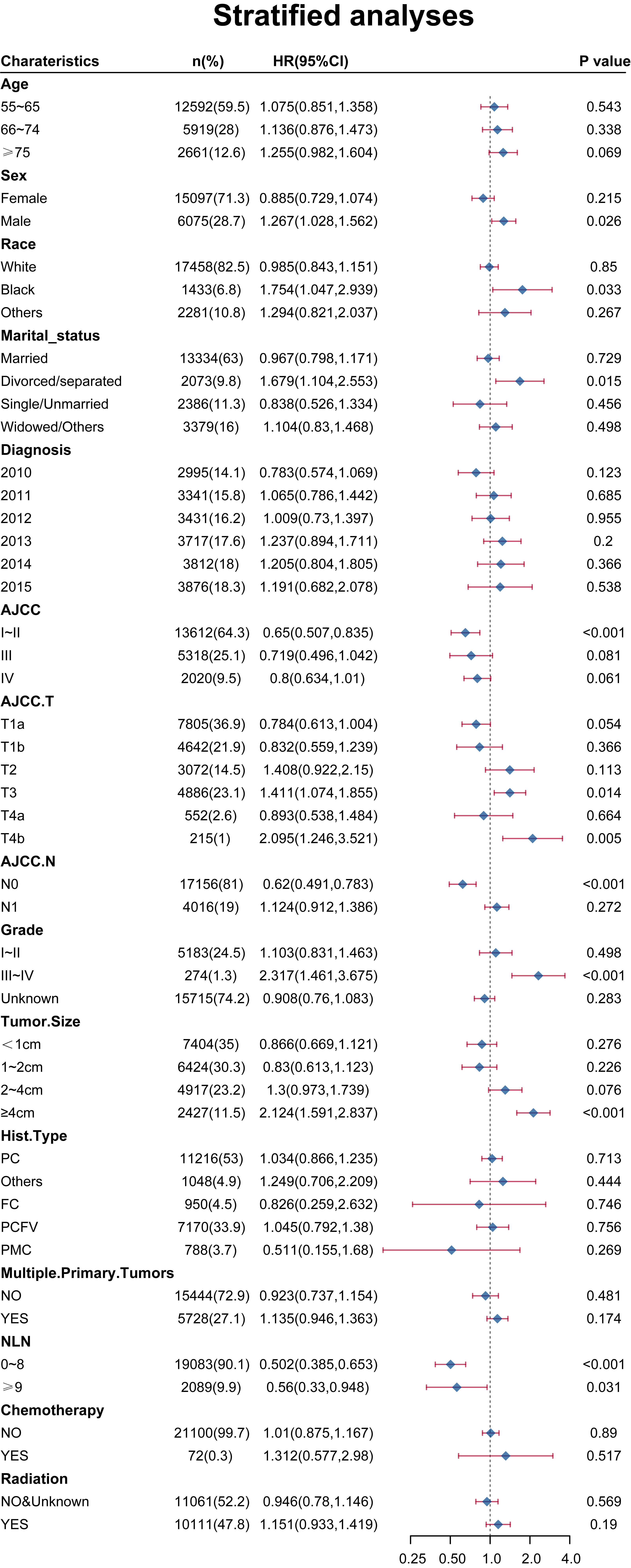

Supplement: Supplementary Figure 2 — Subgroup analyses for LONT. [file Image_2.tif]

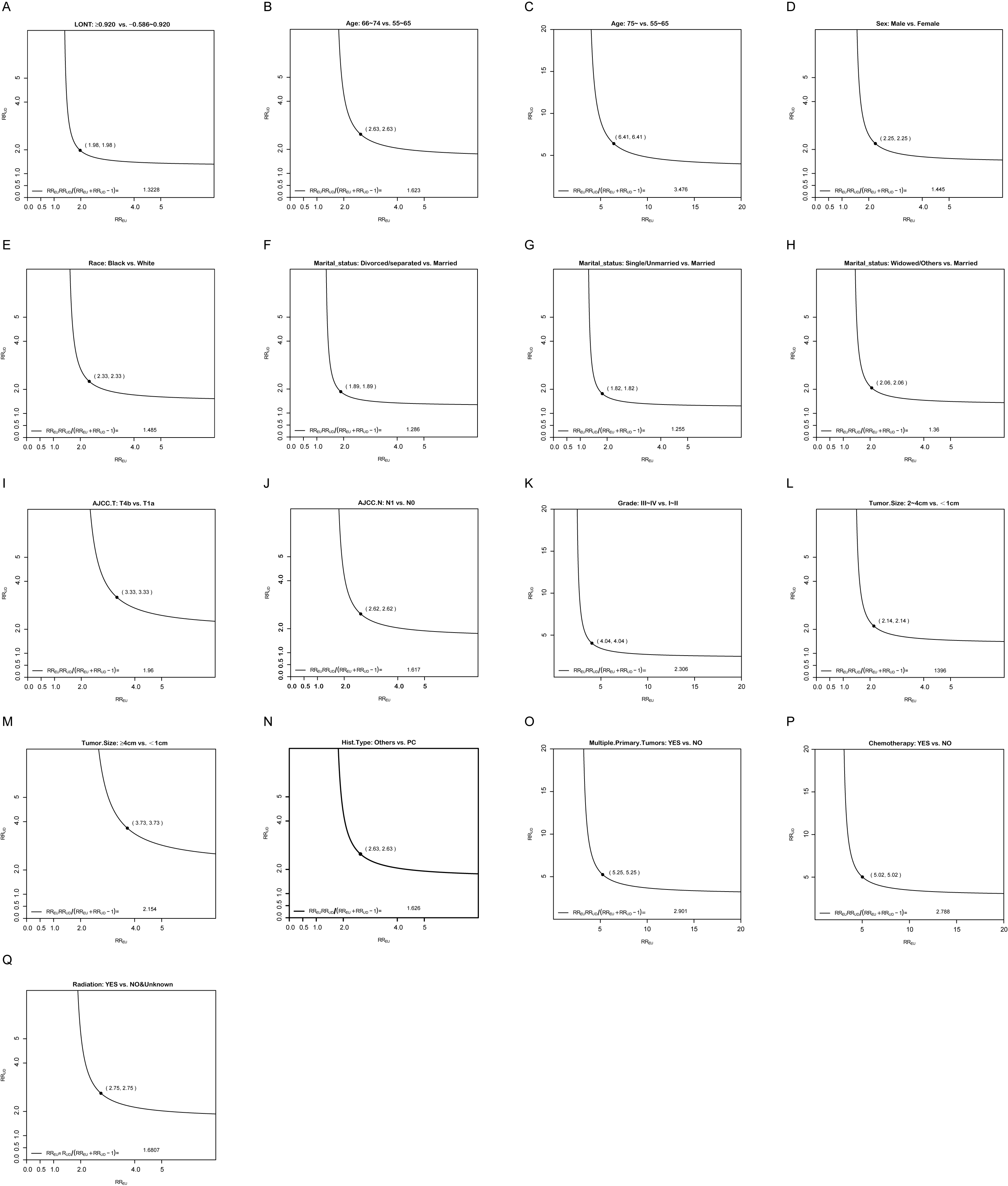

Supplement: Supplementary Figure 3 — E-value of LONT and clinical features in sensitivity analyses. [file Image_3.tif]
